# New methods for the quantification of mixed chimerism in transplantation

**DOI:** 10.3389/fimmu.2023.1023116

**Published:** 2023-01-19

**Authors:** Christophe Picard, Coralie Frassati, Nicem Cherouat, Sandrine Maioli, Philippe Moskovtchenko, Mathilde Cherel, Jacques Chiaroni, Pascal Pedini

**Affiliations:** ^1^ Immunogenetic Laboratory, EFS PACC, Marseille, France; ^2^ CNRS, EFS, ADES, Aix Marseille Université, Marseille, France; ^3^ Immunogenetic Laboratory, EFS AURA, Lyon, France; ^4^ Immunogenetic Laboratory, EFS BRET, Rennes, France

**Keywords:** chimerism, qPCR (quantitative PCR), dPCR (digital PCR), NGS (next generation sequencing), cfDNA (cell-free DNA)

## Abstract

**Background:**

Quantification of chimerism showing the proportion of the donor in a recipient is essential for the follow-up of hematopoietic stem cell transplantation but can also be useful to document an immune tolerance situation after solid organ transplantation. Historically, chimerism has been quantified from genomic DNA, but with technological advances, chimerism from donor-derived cell-free DNA seems particularly relevant in solid organ transplantation.

**Methods:**

The reference method was until recently the short tandem repeat technique, but new innovative techniques as digital PCR (dPCR) and NGS, have revolutionized the quantification of chimerism, such as the so-called microchimerism analysis. After a short review of chimerism methods, a comparison of chimerism quantification data for two new digital PCR systems (QIAcuity™ dPCR (Qiagen^®^) and QuantStudio Absolute Q (ThermoFisher^®^) and two NGS-based chimerism quantification methods (AlloSeq HCT™ (CareDx^®^) and NGStrack™ (GenDX^®^)) was performed.

**Results:**

These new methods were correlated and concordant to routinely methods (r²=0.9978 and r²=0.9974 for dPCR methods, r²=0.9978 and r²=0.9988 for NGS methods), and had similar high performance (sensitivity, reproductibility, linearity).

**Conclusion:**

Finally, the choice of the innovative method of chimerism within the laboratory does not depend on the analytical performances because they are similar but mainly on the amount of activity and the access to instruments and computer services.

## Introduction

The study of hematopoietic chimerism consists of quantitatively distinguishing the population of donor cells from recipient cells in patients after hematopoietic stem cell transplantation (HSCT). The purpose of such analysis is to predict or detect graft rejection following engraftment, early relapse, and graft versus host disease (GVHD) (1). This quantification is performed in different tissues, such as peripheral blood, bone marrow and cells. Various methods have been used as nongenetic methods (fluorescent *in situ* hybridization (FISH) on sex chromosomes and red cell phenotyping) and, more recently, the study of polymorphic genomic markers. Regarding polymorphism, its analysis has evolved considerably over time. Initially, it consisted of the search for restriction fragment length polymorphism (RFLP)-type markers. Then, it involves the sequences called variable number of tandem repeats as minisatellites or variable number tandem repeats (VNTRs) and microsatellites or short tandem repeats (STRs), which interested the molecular biology laboratories in the 2000s. Finally, other markers, such as short insertion/deletion polymorphisms (SIDP) detected by quantitative PCR (qPCR) and more recently by digital PCR (dPCR) and finally by next-generation sequencing (NGS) technology, increased the sensitivity of chimerism quantification, allowing microchimerism (<1%) detection in HSCT.

### RFLP markers

As early as 1985 (2), a technique for studying RFLP of DNA was published. This method consists of looking for variations in the DNA sequence, revealed by the presence or absence of a restriction site. These are sequences specifically recognized and cleaved by a given restriction enzyme. This enzyme is an endonuclease that specifically cuts the two DNA strands at a perfectly defined sequence of 4 to 8 nucleotides, which are generally palindromic. It has been possible to quantify it with a sensitivity on the order of 5 to 20% (3, 4) by the Southern blot method. PCR amplification of specific regions of the Y chromosome (SRY gene) has also been proposed, with sensitivities on the order of 0.01%, but its application has been limited to transplants between donors and recipients of different sexes (5).

### VNTR and STR markers

The main interest of the STR and VNTR markers is that these loci are inherited in Mendelian transmission and are therefore useful for assessing chimerism after HSCT (6).

The first to be used is the VNTR marker, whose analysis allows for increased sensitivity (5 to 10%), use of smaller amounts of DNA, easier DNA preparation, faster turnaround time, elimination of restriction enzymes and radioisotopes, and an overall reduction in cost compared to RFLP markers (7). The method is a PCR amplification of 5-10 VNTR markers revealed by polyacrylamide or agarose gel electrophoresis. The interpretation is based on the different sizes of the amplicons of the donor/recipient couple (7, 8). Despite the simplicity of the VNTR technique, microsatellites (STRs) have become the gold standard for chimerism quantification since the early 2000s. This is partly explained because the repeat regions of many minisatellites are GC rich, because a limitation of available markers renders the technique noninformative in a few cases, and because minisatellite alleles are larger than microsatellite alleles, inducing less technical sensitivity (9).

The STRs correspond to DNA sequences made up of repeated di-tri- or tetra-nucleotide motifs. As the number of repeats varies from one individual to another, these sequences become very informative polyallelic markers (10). Separation and detection of the amplified PCR products is performed more frequently on an Applied Biosystems (ABI) automated DNA sequencer, and the analysis of results is performed using Genescan 2.1 software (ABI) (11, 12).

STR allows to reach a detection sensitivity of between 1 and 5% (**Table 1**). This sensitivity varies from one donor/recipient couple to another due to the more or less favorable allelic configurations. In this case, PCR is not a quantitative technique. However, when amplifying a post-transplant sample, the donor and recipient alleles have similar sequences and are coamplified with the same primers. However, due to its competitive status, the STR method can induce allelic imbalance, allele drop-out and, more frequently, a stutter peak.

**Table 1:** Comparison of STR, qPCR, dPCR and NGS technologies.

	STR*	qPCR	dPCR	NGS
System Description				
Number of markers	12–24	19–48	as qPCR	24 to 202
Need for prior genotyping	Yes	Yes	Yes	No
Adapted to single or series	Yes	Yes	Yes	No
Principe of quantification	Fragments sizes	Quantitative PCR	Partitioning PCR	Sequencing reads
Features				
Quantity of DNA	1-5 ng	150 ng	150 ng	60-100 ng
Sensitivity	1-5%	0.1%	0.05-0.1%	0.1-0.5%
Measurement range	1/5-100%	0.1–20%	0.05/0.1–100%	0.1/0.5–100%
Quantification type	Relative	Relative	Absolute	Relative
Need to replicate	No	Yes	No	No
Workflow				
Total time to deliver results	15h	3 h	90–180 min	24 h
Hands on time	3h	45 min	<15 min	45 min-2 h
Automatable	No	No	Yes	Yes
Ease of use	No	Yes	Yes	Yes
Number of tests/run	1/96	3	1 to 16/24	up to 96/384
Ease of interpreting results	Low**	Low**	Low**	High***
Software included	Yes	Yes	No	Yes

*Historical reference method.

**manual and selection of informative markers.

***identical informative panel markers and automatic calculation.

Under these conditions, the comparison of the intensity of the signal obtained for a specific allele of a donor with that obtained for a specific allele of a recipient makes it possible to evaluate the relative quantity of cells of the recipient by referring to a range of dilutions of DNA of the recipient into that of the donor. In this way, chimerism can be determined quantitatively. The fluorescent labeling of the PCR products obtained for each allele has favored the development of these quantitative measurements and has made it possible to improve their reproducibility, with intermanipulation variation coefficients of less than 10% (11–13) for 5% chimerism.

### SIDP markers by qPCR

Another approach is based on the study of biallelic SIDP. These polymorphisms are less informative than STRs, but they have the advantage of being amenable to specific allele amplification. In addition, a quantitative test based on quantitative real-time PCR, with detection by Taqman^®^ probe, has been developed and adapted to the specific amplification of the recipient’s alleles (14). qPCR is a relative quantitative method that estimates the cycle threshold (Ct), which is inversely proportional to the initial amount of target DNA. Calculation of cycle threshold differences (ΔΔCt) allows calculation of the percentage of chimerism. In this calculation, the post-HSCT hematopoietic DNA Ct is compared to the pretransplant recipient or donor DNA Ct, and the data are normalized with a reference gene Ct. In 2002, Alizadeh et al. (14) first demonstrated that 19 SNP qPCR provided good sensitivity (0.1% recipient DNA per 100 ng DNA). Other studies have repeated this observation with InDel-type polymorphisms (15, 16) with a longer sequence and thus improved discrimination. Today, there are different commercial kits with 10-48 InDel markers depending on the manufacturer. The sensitivity of InDel-based qPCR chimerism analysis has been confirmed at 0.1% (**Table 1**), but some authors observe a sensitivity up to 0.024% depending on the amount of DNA used (from 25 ng to 300 ng) (17). The disadvantages of the qPCR technique are related, on the one hand, to analytical performance and, on the other hand, to methodology. Indeed, the major weakness of qPCR is poor accuracy in the quantification of mixed chimerism (>30%). Moreover, repetitions (duplicates, triplicates) from at least two target probes in each cycle for each target are necessary to provide the most accurate result possible and to control the sensitivity to PCR inhibitors. In addition, the use of pretransplant DNA is necessary, as it is a relatively quantitative method. However, the availability of pretransplant DNA can be a problem when the amount of DNA is limited (cytopenia for recipient or cord blood for donor) or when the chimerism analysis has been repeated several times.

### SIDP markers by digital PCR

The principle of digital PCR (dPCR) was discovered by Sykes et al. in 1992 (18). This is a method for absolute quantification of target nucleic acids. Quantification by dPCR is based on partitioning the sample into thousands of compartments that are independent PCR nanoreactors. The nucleic acid arrays are randomly distributed in these compartments with a statistical distribution that follows Poisson’s Law to have only one or at most a few copies of nucleic acids in each compartment. The interest of this system, compared to classical PCR, is to be able to search for a target of low allelic abundance among other more represented targets. Thus, the rare target is individualized in a compartment, either alone if the initial concentration is low (or if the number of compartments is very large) or perhaps mixed with some other copies present in much lower abundance than in the initial sample. Once the distribution of the nucleic acids in each compartment is performed by filling the microchambers or droplets, each of them will then constitute an individual nanoreactor for the specific amplification of the target of interest, if it is present. At the end of the reaction (end-point analysis), the number of positive compartments (presence of a fluorescent signal reflecting amplification) and negative compartments (absence of a fluorescent signal above the background reflecting the absence of amplification) are counted, thus defining a digital signal of the “all or nothing” type (0 or 1). This digital signal is opposed here to the analog signal of an exponential type used in real-time qPCR. This droplet count is used to deduce the target DNA copy number using the formula λ = - ln (1 - *p*), where λ is the average copy number per compartment and p is the ratio of the number of positive compartments to the total number of compartments.

The main advantages of this technology are (i) stability of sensitivity (less to 0.1%) and the increase of reproducibility, allowing the detection of a rare event, (ii) the limited influence of classical PCR inhibitors (SDS, heparin, ethanol, melanin, hemoglobin) due to the very low reaction volumes and the partitioning of the inhibitors whose concentration is generally low, (iii) the possibility to obtain an absolute quantification of the copy number without the need for an external reference sample or a calibration curve, and (iv) the possibility of multiplexing the amplifications thanks to compartmentalization of the DNA copies without creating competition between the targets.

In contrast, the main limitation of this technology is a performance that decreases when the amount of DNA increases, with a limit specific to each application, in particular according to the number of compartments available.

### Current dPCR systems

Currently, there are various platforms of dPCR that are different according to the mode of partitioning. It can be partitioned on a solid support called microchambers (chamber dPCR or cdPCR)) or in oil/water emulsions called droplets (droplet dPCR or ddPCR).

In 2006, Fluidigm^®^ launched the Biomark™ system, which was considered the first commercial dPCR system platform. The BioMark™ system was capable of producing 765 chambers with a volume of 6 nL. The development of microfluidic systems has permitted a new generation of dPCR by allowing the creation of nanodroplets instead of microchambers. This technology was first commercialized by QuantaLife, Inc. as droplet digital PCR (ddPCR) technology in 2011 with a QX100 system. Bio-Rad Laboratories, Inc. acquired QuantaLife, Inc. and offered the QX200 and QXONE dPCR instruments. The ddPCR platform (Bio-Rad^®^) allows the generation of up to 20,000 droplets. This advance in droplet compartmentalization technology enabled Stilla^®^ to launch the Naica™ dPCR platform, known as Crystal Digital PCR™, in 2016. This system divides samples into a 2D array of thousands of individual droplet crystals, each with its own reaction compartment.

In 2013, Formulatrix’s Constellation dPCR instrument was launched, which integrates partitioning, thermal cycling, and imaging into a single fully automated instrument. However, it was not until 2019 that Formulatrix was acquired by Qiagen^®^ (Hilden, Germany) and reintroduced the technology as QIAcuity™ in 2020. The QIAcuity dPCR system offers two sample compartmentalization systems for different applications. For high-throughput applications, such as gene expression analysis, experiments can accommodate 96 samples divided into 8,500 partitions per sample, while for sensitive applications, such as rare mutation detection, plates dividing 24 samples into 26,000 partitions per sample can be used. That is why three different features of QIAcuity™ dPCR are available on the market, i.e., QIAcuity One, Four, and Eight. Finally, among the first to engage in the development of dPCR, ThermoFisher Scientific^®^ with QuantStudio dPCR series also uses cdPCR technology for its QuantStudio 3D dPCR and QuantStudio 12 K Flex systems. In 2022, the company launched the QuantStudio Absolute Q system. This new system allows the creation of more than 20,000 partitions in a 12-sample plate.

### dPCR and chimerism

dPCR is able to absolutely quantify a specific target DNA sequence of a recipient or donor with greater accuracy than qPCR and without the need for a calibration curve (19). Furthermore, Stahl et al. (20) considered dPCR as a combination of STR-PCR and qPCR because this technology provides both good sensitivity (qPCR) and high precision and reproducibility (STR-PCR). Our and other authors showed that digital PCR (Bio-Rad^®^) and Crystal digital PCR™ had a similar sensitivity (ie, 0.1%), a range of analysis from 50% to 0.1% (**Table 1**), and reproducibility with a CV <20% (21, 22).

Thus, dPCR is the technology of choice for the determination of both low or micro chimerism and mixed chimerism in a reproducible way and with absolute quantification. Finally, dPCR can be used as an indicator for minimal residual disease (MRD) in the hematology field (19).

Nevertheless, dPCR is characterized by significant disadvantages. First, Santurtún et al. (23) reminded us that a genotyping step by conventional PCR upstream of dPCR is necessary to select informative markers. Second, depending on the chimerism, which is unknown before the analysis, the amount of DNA introduced in the system can be either insufficient to have good sensitivity (case of low chimerism) or too high with saturation of the dPCR system to have good accuracy (case of mixed chimerism) (20). Additionally, some authors consider dPCR expensive because the cost per well is higher than that of STR-PCR. Nevertheless, multiplexing, automation, and lack of control of the previous collection can overcome this financial problem.

At present, the literature on the study of chimerism mainly concerns ddPCR *via* BioRad systems (19–21, 23–25) (QX platform) and *via* the Stilla system (22) (Naica platform). No comparative study has investigated the study of chimerism on other dPCR systems. In this context, our team has already studied ddPCR systems (21, 22) (QX and Naica platforms).

### Next-generation sequencing

With high sensitivity, NGS allows the simultaneous sequencing of millions of DNA fragments. These reads were then assembled by aligning them to the human reference genome using bioinformatics tools. Depending on the end goal (e.g., targeted gene sequencing, exome sequencing, or even whole genome sequencing), some NGS technologies/platforms are more suitable than others. NGS can work in multiplex so a large number of SNPs can be studied simultaneously (1). NGS technology has revolutionized the field of genomics, and applications for clinical diagnosis have been numerous. In the context of chimerism monitoring, the possibility of working in a panel offers an unprecedented opportunity to distinguish two or more different genomes. NGS provides qualitative and quantitative data. Quantitative data depend on the depth of sequence data collected on each sample and on the quality of the target to be exposed. For samples with a lower abundance target, many more sequence reads are required to achieve accurate quantification (26). In chimerism monitoring (**Table 1**), different SNP marker panels were initially developed to be analyzed by NGS. However, Vives et al. (27) proved that STR and Indel markers can also be analyzed with these new high-throughput sequencing technologies to detect chimerism. Currently, NGS has shown close concordance with historical results from qPCR, STR and dPCR analysis (22). Their sensitivity has reached 0.1% to 0.5% and allows quantification of mixed chimerism (17, 26). Existing panels are characterized by numerous SNPs selected to obtain many informative markers for each HSCT pair and are located in different regions of each chromosome. The advantage of marker panels is that they do not require upstream marker selection and manage the fastidious logistics of single markers in the laboratory. Therefore, interestingly, genotyping of genetic markers of donor-recipient pairs and quantification of chimerism are performed in the same protocol, which takes between 24 and 48 hours, depending on the kits. Today, several commercially available kits offer an NGS method for chimerism as a complete workflow solution for laboratories, combining a reliable testing process with specially designed analysis software. In this context, our team has already studied the Devyser^®^ panel (Devyser Chimerism NGS) (22).

In total this work has two purposes. First, to establish a comprehensive overview of reference and innovative technologies for the quantification of chimerism. Secondly, a study of chimerism quantification for two new digital PCR systems (QIAcuity™ dPCR (Qiagen^®^) and QuantStudio Absolute Q (ThermoFisher^®^) and two NGS-based chimerism quantification methods (AlloSeq HCT™ (CareDx^®^) and NGStrack™ (GenDX^®^)) was performed in comparison of QX200 ddPCR (Biorad^®^) using the same procedure as previously described (22). In brief, a range of artificial chimerism and samples (whole blood, bone marrow and cell sorting) from patients were used to define the analytical performance of each method (correlation, concordance and reproducibility).

## Materials and methods

### Biological samples

Artificial chimeric samples were prepared from the donor samples of 2 healthy subjects (1 male and 1 female). To study the linearity of measurement, we prepared DNA dilutions of the male and female samples at concentrations of 50%, 10%, 1%, 0.5%, and 0.1%. This system was qualified by qPCR and ddPCR techniques. Twelve quality control samples with known targets for qPCR, dPCR and NGS techniques were from the SFHI program (Société Francophone d’Histo-compatibilité et Immunogénétique, External Proficiency Testing). A total of 38 samples from 5 patients with hematologic malignancies treated with allogeneic HSCT were included in this study. Samples came from 3 major immunogenetic laboratories of the Etablissement Français du Sang (EFS), i.e., 2 adult patients from Lyon, 1 pediatric patient from Rennes and 2 pediatric patients from Marseille. The initial diseases were immune deficiency (ID) and, more precisely, granulomatosis disease in 1 pediatric patient, thalassemia (Th) in 1 pediatric patient, acute myeloid leukemia (AML) in 2 adult patients and unknown hematological disease in 1 pediatric patient. For the samples, 28 were from whole blood, 5 were from bone marrow, and 5 were from cell sorting (1 CD15, 1 CD33, 1 CD34, 2 CD3). The CD15, CD33 and CD3 cells were purified from whole blood from different donors on beads from the EasySep Human Whole positive selection kit (Stem Cell technologies, Vancouver, BC, Canada), according to the supplier’s recommendations. The CD34 cells were enriched from whole blood by MACSprep™ Chimerism CD34 MicroBead Kit (Milteny I Biotec) utilizing MACS^®^ Technology, following the recommendation’s manufacturer. Flow Cytometry assessed the percentage of different subpopulations cells.

The chimerism of each sample had been previously quantified by EFS national public market techniques, qPCR analysis using QTrace kits (JETA^®^ Molecular, Utrecht, Netherlands) and NGS analysis using AlloSeq HCT kits (CareDx^®^, Brisbane, CA). In addition, chimerism was previously assessed by ddPCR (Bio-Rad^®^, Hercules, CA) by a method developed (21, 22) using KMRtrack kits (GenDx^®^, Utrecht, Netherlands).

All donors and patients provided written informed consent, after which the clinical data were collected from each patient. The signed informed consent forms were recorded as a part of the patient’s clinical record.

### DNA isolation methods

The genomic DNA of the donors (peripheral whole blood) and recipients (whole blood, cell sorting isolation, and bone marrow) was isolated using the QuickGene 610 L nucleic acid isolation system (Kurabo Industries, Osaka, Japan) or the Chemagic automation system (Perkin Elmer, Waltham, MA). The DNA concentration and purity were checked by absorbance measurement using an ND-One spectrophotometer (Nanodrop Technologies, Wilmington, DE) and fluorometric quantitation (Qubit Technologies, ThermoFisher^®^). All the DNA used in the study had absorbance ratios of A260/A280 >1.8 and A260/A230 >1.8. The DNA samples were stored at -40°C for <48 months before use in the experiments.

### Chimerism analysis by cdPCR

The principle and technical characteristics of the two cdPCR systems tested in this work (QuantStudio Absolute Q and QIAcuity) and those of the two other digital PCRs (ddPCR QX200 and Crystal digital PCR) previously published (22) are described in **Table 2**.

**Table 2:** Comparison of dPCR methods for chimerism analysis. +++: very easy, ++: easy, +: less easy.

	QX200 System	Crystal Naica System	QIAcuity	Absolute Q
System description				
Number of modules	3*	2	1	1
Instrument dimension (cm)	Generator: 28 x 36 x 13	Geode: 35 x 37 x 29	38 x 45 x 65	62 x 54 x 60
	Reader: 66 x 52 x 29	Prism3: 44 x 34 x 21		
On-board computer	No	No	Yes	No
Partitioning type	Droplet (oil emulsions)	Droplet (« crystal »)	Solid (nanoplate)	Physical (microchambers)
Number of partitions	20,000	20,000–30,000	8,500 or 26,000	20,480
Fluorescent channels	2–4	3–6	6	5
Types of markers/Fluorescent molecules	SNP-Indel/TaqMan-EvaGreen	SNP-Indel/TaqMan	SNP-Indel/TaqMan-EvaGreen	SNP-Indel/TaqMan
Need for prior genotyping	Yes by qPCR	Yes by qPCR	Yes by qPCR	Yes by qPCR
Adapted to single or series	1-96 samples/run	12 to 48 samples/run	8-96 samples/run	4-16 samples**
Multitransplants possible	Yes	Yes	Yes	Yes
Compatible with cfDNA	Yes	Yes	Yes	Yes
Features				
DNA quantity (for sensitivity 0.1%)	150 ng	150 ng	150 ng	150 ng
Sensitivity	0.1%	0.1%	0.1%	0.1%
Measurement range	0.1 to 100%	0.1 to 100%	0.1 to 100%	0.1 to 100%
Type of quantification	Absolute (cp/µL)	Absolute (cp/µL)	Absolute (cp/µL)	Absolute (cp/µL)
Need for replicates	No	No	No	No
Workflow				
Total time (min)	180	150	90	90
Hands on time (min)	30	15	<15	<15
Technical steps	6	4	3	3
Ease of use	+	++	+++	+++
Software				
Security/Tracability (21 CFR Part 11)	Yes	not reported	Yes	Yes
Patients database	No	No	No	No
Automatic calculation	Yes	No,	No	No
Easy to use	+	++	+++	+++
Custom adjusted cutoff	Yes	Yes	Yes	Yes
Data export	Yes	Yes	Yes	Yes
Report edition	Yes	Yes	Yes	Yes

*Including PCR thermocycler.

**Ability to reuse unused areas of the plate.

### QIAcuity™ dPCR method

The QIAcuity is designed as a walk-away instrument that integrates and automates all plate processing steps. Only plate preparation must be performed manually before starting the run. In instrument, the first step consist in filling the plate’s microchannels and partitions with the input volume in the wells. The second step is a high-accuracy plate thermocycler that performs the polymerase chain reaction. The cycling profile can be set in the QIAcuity Software Suite or the instrument software. The final step is the image acquisition of all wells. The user can select the detection channels in the experimental setup. The partitions that have a target molecule inside emit fluorescence light and are brighter than those without a target. Depending on the configuration of the instrument (2plex/5plex), there are up to 2 or 5 selectable detection channels. An additional channel is used for detecting the base fluorescence of the master mix to determine the exact number of filled partitions and normalization of fluorescence data.

Chimerism was quantified using the QIAcuity One, 5plex Device with a 5-color target multiplexing capability with several emission wavelengths, 518 to 548 nm (FAM, EvaGreen^®^), 550 to 564 nm (HEX, VIC, JOE), 580 to 606 nm (TAMRA, ATTO 550), 611 to 653 nm (ROX, Texas Red) and 654 to 692 nm (Cy5), with ROX dye used for reference. For 1 sample and a reaction volume of 44 µL, we used 11 µL of 4x Probe PCR Master Mix (Qiagen^®^), 11 µL of 4x KMRtrack FAM, 2.2 µL of 20x RPP30 gene control HEX, 13.8 µL of RNase-free water and 6 mL of 25 ng/mL DNA (150 ng). The plate used is not reusable; it has a format of 3x8 wells and has a barcode for traceability. The 44-µL PCR mixture was pipetted into each of the inlet ports of the QIAcuity Nanoplate 26k 24 wells, which was then resealed with QIAcuity Nanoplate Seals using a roller. Finally, the plate was installed in the instrument, and all steps (priming, cycling and imaging) were performed automatically. The PCR program had to be adapted for chimerism analysis: 95°C for 2 min, followed by 45 cycles of 95°C for 15 s and 58°C for 1 min. Image acquisition was performed using the following exposure times: green signal, 400 ms with the gain at 4 and yellow signal, 300 ms with the gain at 3. The time of the complete procedure was 2 h. Data were analyzed using the QIAcuity 2.0.20 software suite, which automatically calculates and defines the optimal threshold for discrimination between positive and negative partitions. This threshold can be modified manually. Quantification of donor or recipient chimerism is obtained by the ratio of the number of partitions to the informative target/nonpolymorphic target.

### QuantStudio Absolute Q method

The QuantStudio Absolute Q Digital PCR system integrates all the steps required for dPCR, including digitalization, scanning, thermal cycling and data acquisition, into one instrument. The reaction mixture was pipetted into the MAP plate, and isolation buffer was added. The Applied Biosystems™ QuantStudio™ Absolute Q™ MAP16 Digital PCR consumable is used with the QuantStudio Absolute Q Digital PCR system. The system is powered by microfluidic array plate (MAP) technology that enables consistent and automated separation of the sample into 20,480 microchambers, leading to a <1% coefficient of variation (CV).

Chimerism was quantified using the QuantStudio Absolute Q, 5plex Device with a 4-color target multiplexing capability with several emission wavelengths, including a peak at 520 nm (FAM), peak at 560 nm (HEX, VIC), peak at 589 nm (ABY), peak at 625 nm (ROX) and peak at 684 nm (JUN, Cy5), with ROX dye used for reference. For 1 sample and a reaction volume of 10 µL, we used 2 µL of 5x QuantStudio Absolute Q DNA Master Mix (ThermoFisher^®^), 2.5 µL of 4x KMRtrack FAM, 0.5 µL of 20x RPP30 gene control HEX and 5 µL of 30 ng/mL DNA (150 ng). MAP16 is partially usable and reusable; it has a format of 4x4 wells and has a barcode for traceability. Nine microliters of the PCR mixture were pipetted into each of the inlet ports of MAP16, and isolation buffer was added to the system. Finally, the plate was installed in the instrument, and all steps (digitization, thermal cycling, and data acquisition) are performed automatically. The PCR program did not need to be adapted for chimerism analysis: 96°C for 10 min, followed by 40 cycles of 96°C for 5 s and 60°C for 15 s. Image acquisition was performed using manufacturing settings. The time of the complete procedure was 1 h and 30 min. Data were analyzed using QuantStudio Absolute Q Digital PCR software 6.1.0, which automatically calculates and defines an optimal threshold for discrimination between positive and negative partitions. This threshold can be modified manually. Quantification of donor or recipient chimerism is obtained by the ratio of the number of partitions to the informative target/nonpolymorphic target.

### Chimerism analysis by NGS

The principle and technical characteristics of the two NGS systems tested in this work (CareDX^®^ (AlloSeq HCT) and GendX^®^ (NGStrack)) and those of the Dvysr^®^ (Devyser Chimerism) method previously published (22) are described in **Table 3**.

**Table 3:** Comparison of NGS methods for chimerism analysis. +++: very easy, ++: easy, +: less easy.

	Devyser Chimerism NGS	AlloSeq HCT	NGStrack
System description			
Number of markers	24	202	35
Types of markers	Indel	SNP	Indel
Genotyping for all contributors	Yes	No *	Yes
Pool with other libraries	No	No	Yes **
Number of PCRs/sample	2	1	7
Flow cell type	V2 micro or nano 300 or V3 150	V3 150	V2 standard, micro or nano 150
Multitransplants possible	Yes. Up to 3 genotypes	Yes. Up to 3 genotypes	No ***
Illumina sequencers	iSeq, MiSeq, MiniSeq	MiSeq, MiniSeq	MiSeq, iSeq, MiniSeq, NextSeq
Ergonomics	High, simple technique	High, simple technique	Intermediate, 7 mixes/sample
Reagent storage	4 and -20°C	4 and -20°C	-20°C
CE-IVD	Yes	Yes	No
Compatible with cfDNA analysis	Yes	Yes	Not communicated
	existing kit for dd-cfDNA	existing kit for dd-cfDNA	
Features			
DNA quantity	60 ng	10 ng	Genotyping: 20-100 ng/mix Monitoring: 20-40 ng/mix
Sensitivity	0.1%	0.22%	0.5%
Measurement range	0.1 to 100%	0.22 to 100%	0.5 to 100%
Type of quantification	Based on ratio short:long reads of the Indel.	Based on VAF % of SNP	Based on ratio short:long reads of the Indel
Need for replicates	No	No	No
Workflow			
Total time (according flowcell)	Results in less than 24 h or ~ 48h (V2 standard)	Results in less than 24 h	Results in less than 24 h
Technical steps	7	5	5
Hands on time	~ 45 min	<90 min	~ 2 h
PCR runs	1h45 + 1h55	1h30	1h40
Number of purifications	1	2	1
Ease of use	++	+++	++
Number of patients	Up to 96	Up to 48	384 genotyping/32 monitoring
Software			
Security/Tracability (21 CFR part 11)	Not reported	Yes	No
Patients database	Yes	Yes	No
Automatic calculation	Yes	Yes	Yes
Easy to use	+++	+++	++
Quality criteria (including contamination)	Yes	Yes	No ***
Manually selection of markers	Yes	No	No ***
Interpretation time (upload fastq/one sample analysis)	10–30 min/30 sec	10–30 min/30 sec	10–30 min/30 sec
Data export (format)	PDF, XML, Excel	CSV, TSV, PDF	PDF
Report edition	Yes	Yes	No
Graph of follow-up	Yes	Yes	Yes

*for N genomes samples: N-1 only, performed only once; ** (HLA typing, HLA loss); *** possible in further update TRKengine release 1.3 (Q4 2022); dd-cfDNA, donor derived cfDNA, VAF, variant allelic frequencies.

### AlloSeq HCT method

The AlloSeq HCT is a targeted, next-generation sequencing (NGS) assay that utilizes differences in single nucleotide polymorphisms (SNPs) to measure the amount of recipient and donor-derived DNA present in a posttransplant sample. Each reagent kit contains all reagents required for the multiplex amplification of 202 loci for up to 96 samples. Each amplicon covers a unique SNP, which is spread across all human autosomal chromosomes. The complete sequencer-ready amplicon is generated in a single amplification step and contains the target region, dual sample-specific indices and flow-cell adapters. Once all sample libraries were generated and pooled, the resulting library was subjected to magnetic bead clean-up for the removal of primers followed by quantitation. The Illumina MiSeq workflow was then followed to prepare the library for sequencing. The sequencing reaction was performed using the MiSeq v3 Reagent kit using 75 paired-end reads and dual barcoding. A modified sample sheet was prepared for the sequencing reaction, and data analysis of the fastq files was performed using AlloSeq HCT Software.

The AlloSeq HCT chimerism study is possible with a kit of 24 or 48 (plate with precoated indices) samples. For one sample, the reaction volume was 40 µL with 4 µL of reverse index primers, 4 μL of direct index primers, 13 µL of AlloSeq HCT PCR Mix, 0.8 µL of AlloSeq HCT PCR Enzyme, 2.2 µL of AlloSeq HCT SNP Primer Pool and 16 µL of DNA at 0.625 ng/μL (10 ng input). The unique amplification protocol couples both amplification and indexing into a single reaction, limiting user hands-on time and manipulation errors. The thermocycling protocol is unique and corresponds to 98°C for 3 min, 8 cycles of 96°C for 15 s + 70°C for 5 s + 57°C for 60 s + 72°C for 30 s, followed by 12 cycles of 96°C for 15 s and 72°C for 60 s, 72°C for 2 min and 10°C. There was then a double purification step on the beads and finally a denaturation step with 0.2 N sodium hydroxide for the sequencing reaction, which was performed using the MiSeq v3 reagent kit for 150 cycles. The total delivery time was approximately one day, with 2 h of hands-on time and 17 h of NGS sequencing. Data were analyzed using AlloSeq HCT v2 software, which automatically calculates chimerism values and has many alarm QC metrics, some of which are blocking and will prohibit clear display of potentially incorrect results. Genotyping is determined by the % variant allele frequency (VAF) calculation for each marker using the number of reads classified as “reference” (Ref) and “alternative” (Alt) of the reference sequence hg19 (human genome 19; Genome Reference Consortium). The %VAF is calculated as follows: Ref reads/(Ref reads + Alt reads). Pretransplantation samples are expected to have a %VAF close to 0% (-/-), 50% (+/-), or 100% (+/+). For monitoring a marker is informative if the recipient and donor are homozygous for opposite genotypes or if the recipient is heterozygous and the donor is homozygous (+/+ versus -/- or -/- versus +/+ and +/- versus +/+ or -/-), respectively. Multiple samples can be analyzed together, and the analysis time for each sample is 30 s. The software remembers the pretransplant genotyping information, so this only must be done once. Contamination and sample mix-ups are automatically detected. The software allows for storage of data by patient and allows for tracking graphs based on dates, sample types and therapeutics.

### NGStrack method

NGStrack consists of reagents for genotyping both recipient and donor nonchimeric samples. Subsequently, these assays were used to quantify chimerism levels in a mixed sample between the same recipient and donor. Both applications make use of NGS. NGStrack is designed to amplify biallelic indel markers using human genomic DNA specimens. During amplification, target amplicons are simultaneously barcoded. Subsequently, the barcoded amplicons are pooled separately for genotyping and monitoring and subjected to a cleanup step, generating a sequence-ready library to be sequenced on an Illumina sequencing device. A single genotyping experiment on nonchimeric recipient and donor samples should be performed to determine informative markers. Based on this information, future (mixed) samples can be tested to assess the level of chimerism between recipient and donor(s). By combining the data obtained from multiples of these posttransfer samples, the chimeric status of the obtained samples can be monitored.

The NGStrack chimerism study was performed with a 96-well plate but for 12 samples because 7 mixes were needed for one sample. For one mix, the reaction volume was 20 µL with 2 µL of TRK Mix, 4 μL of TRKenzyme, 8 µL of resuspended IndX, 4 µL of nuclease-free water, and 2 µL of DNA (at 10-50 ng/μL for genotyping, at 10 ng/µL for monitoring). The amplification protocol was unique and corresponded to 95°C for 5 min, 35 cycles of 95°C for 15 s + 62°C for 30 s + 72°C for 1 min, followed by a cooling step at 15°C. There was then a purification step on beads at a 0.6X proportion and finally a denaturation step with sodium hydroxide for the sequencing reaction, which was performed using MiSeq V2 sequencing reagents. The total delivery time was approximately one day, with 2 h of hands-on time and 19 or 24 h of NGS sequencing according to the flowcell (micro or standard). Data were analyzed using TRKengine software, which automatically calculates chimerism values. Quantification based on ratio short:long reads of the Indel. Genotyping is homozygous if marker between 0-10%/90-100% short/long and heterozygous if marker between 40 and 60% short/long. For monitoring, a marker is informative if the recipient and donor are homozygous for opposite genotypes or if the recipient is heterozygous and the donor is homozygous (+/+ versus -/- or -/- versus +/+ and +/- versus +/+ or -/-), respectively. The software allows for storage of data by patient and allows for tracking graphs based on dates and sample types.

### Statistical analysis

The square of the Pearson correlation coefficient (R2, P value) was computed to determine the replicability of the system. A Bland–Altmann plot was used to visualize the grade of concordance between the results of chimerism obtained by cdPCR and NGS methods. Because of its reliability over a large range of measurements, ddPCR was taken as a reference technique.

## Results

### Comparison of dPCR automates for chimerism quantification

**Figure 1:**
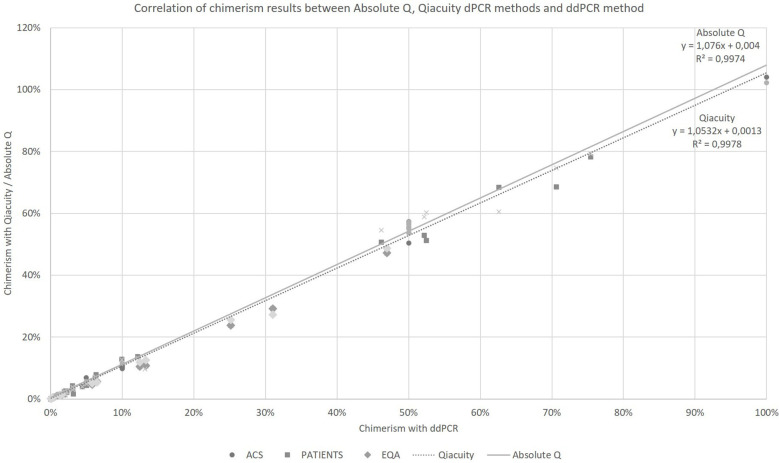
Correlation of chimerism results between Absolute Q, Qiacuity dPCR and ddPCR method (reference method). ACS. Artificial Chimeric Samples; EQA. External Quality Assessment.

Taking into account all the points (details in **Supplementary Data**), regardless of the type of collection (artificial chimerism, patient or EQA) and the tissue type (blood, bone marrow or sorted cells), the Qiacuity and Absolute Q dPCR techniques were correlated with the ddPCR technique (r²=0.9978 and r²=0.9974, respectively, **Figure 1**) over the entire range of measurement from 0.1% to 100%, and using the same KMRtrack probes of interest, the sensitivity of each technique was at least 0.1%.

**Figure 2:**
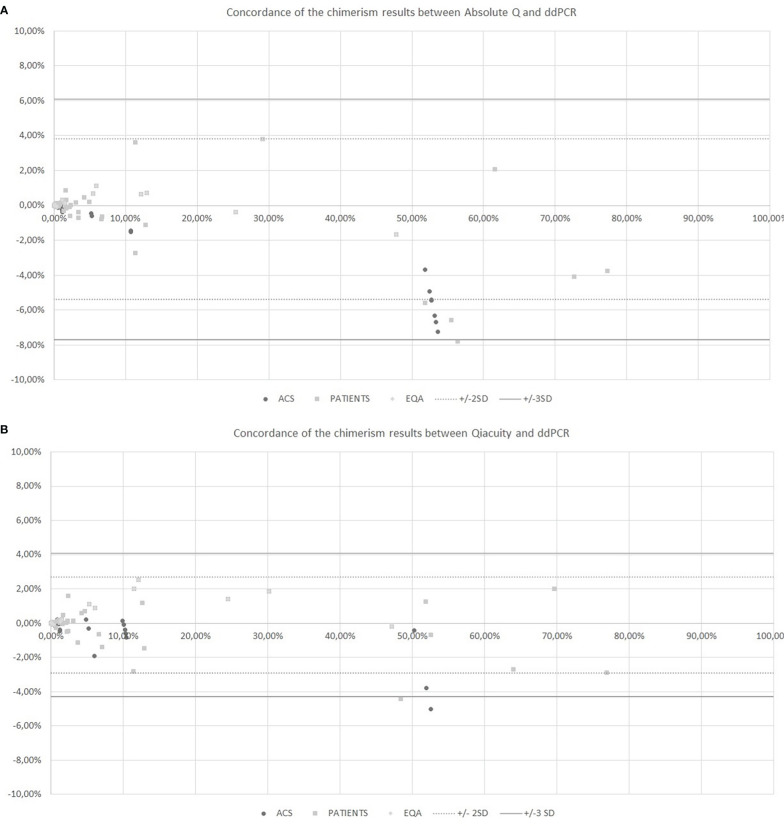
Concordance of the chimerism results between Absolute Q and ddPCR **(A)** and between Qiacuity and ddPCR **(B)** using the Bland-Altman plot. The Bland-Altmann plot includes the ± 2 standard deviation value (dotted lines) and ±3 standard deviation value (solid lines). ACS. Artificial Chimeric Samples; EQA. External Quality Assessment.

The chimerism results from dPCR Qiacuity and the Absolute Q dPCR techniques were correlated and concordant with those from the ddPCR technique (**Figure 2**). On a Bland-Altman plot, only one outlier (< -3 SD) was observed for dPCR Qiacuity, but this was a point of mixed chimerism and the distribution was less scattered than that observed for Absolute Q. Finally, Absolute Q tended to under-estimate chimerism on the mixed chimerism range. Furthermore, all chimerism results from EQA samples tested in both dPCR Qiacuity and dPCR Absolute Q techniques were consistent with the target results.

**Figure 3:**
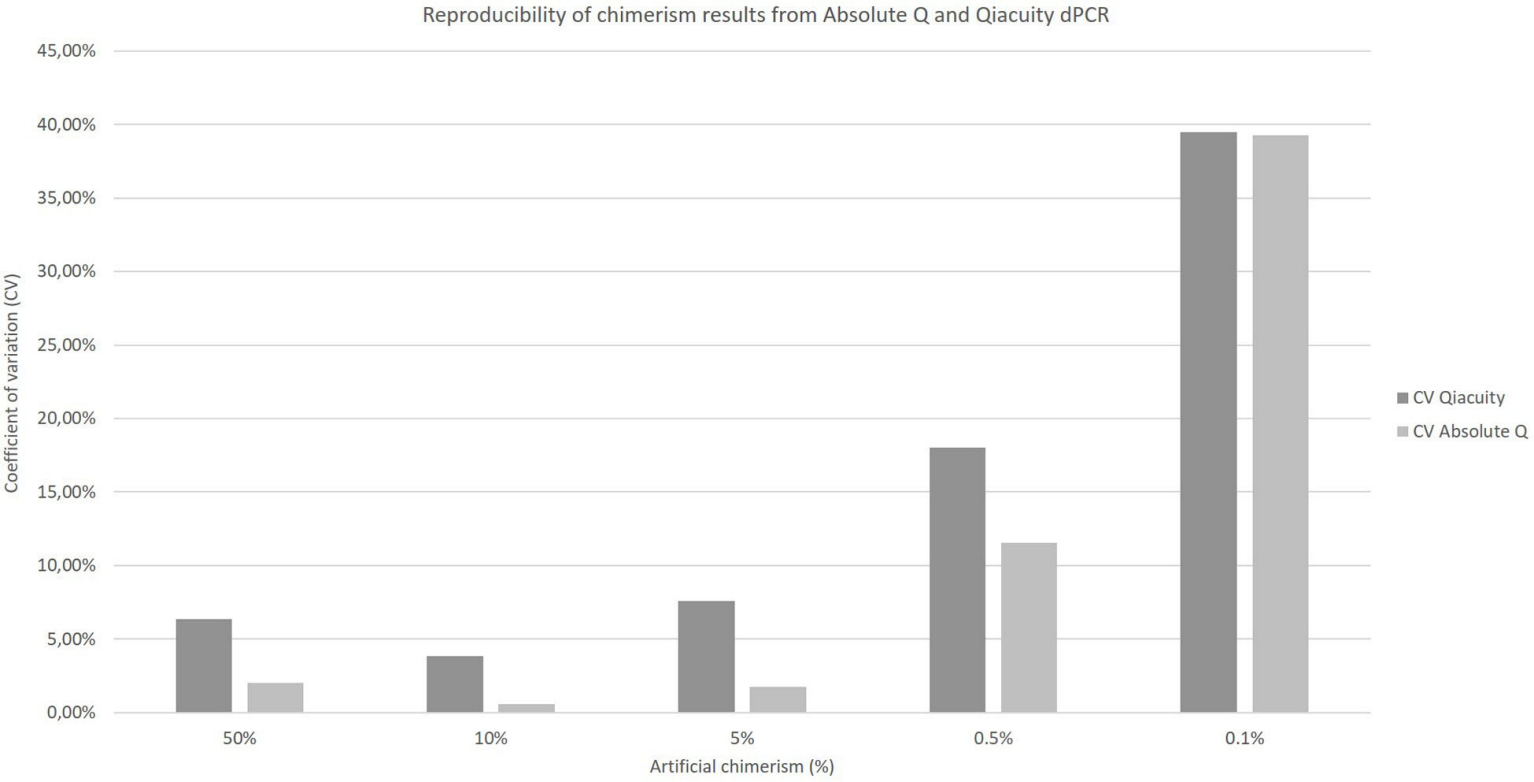
Reproducibility of chimerism results from Absolute Q and Qiacuity dPCR. Each chimerism percentage (50%, 10%, 5%, 0.5%, 0.1%) was tested on at least three different experiments to calculate the coefficient of variation of each method.

The coefficients of variation of reproducibility for both dPCR Qiacuity and dPCR Absolute Q techniques are shown in **Figure 3**. The coefficients of variation for the dPCR Absolute Q technique were lower except for the 0.1% value.

### Comparison of two NGS methods for chimerism quantification

**Figure 4:**
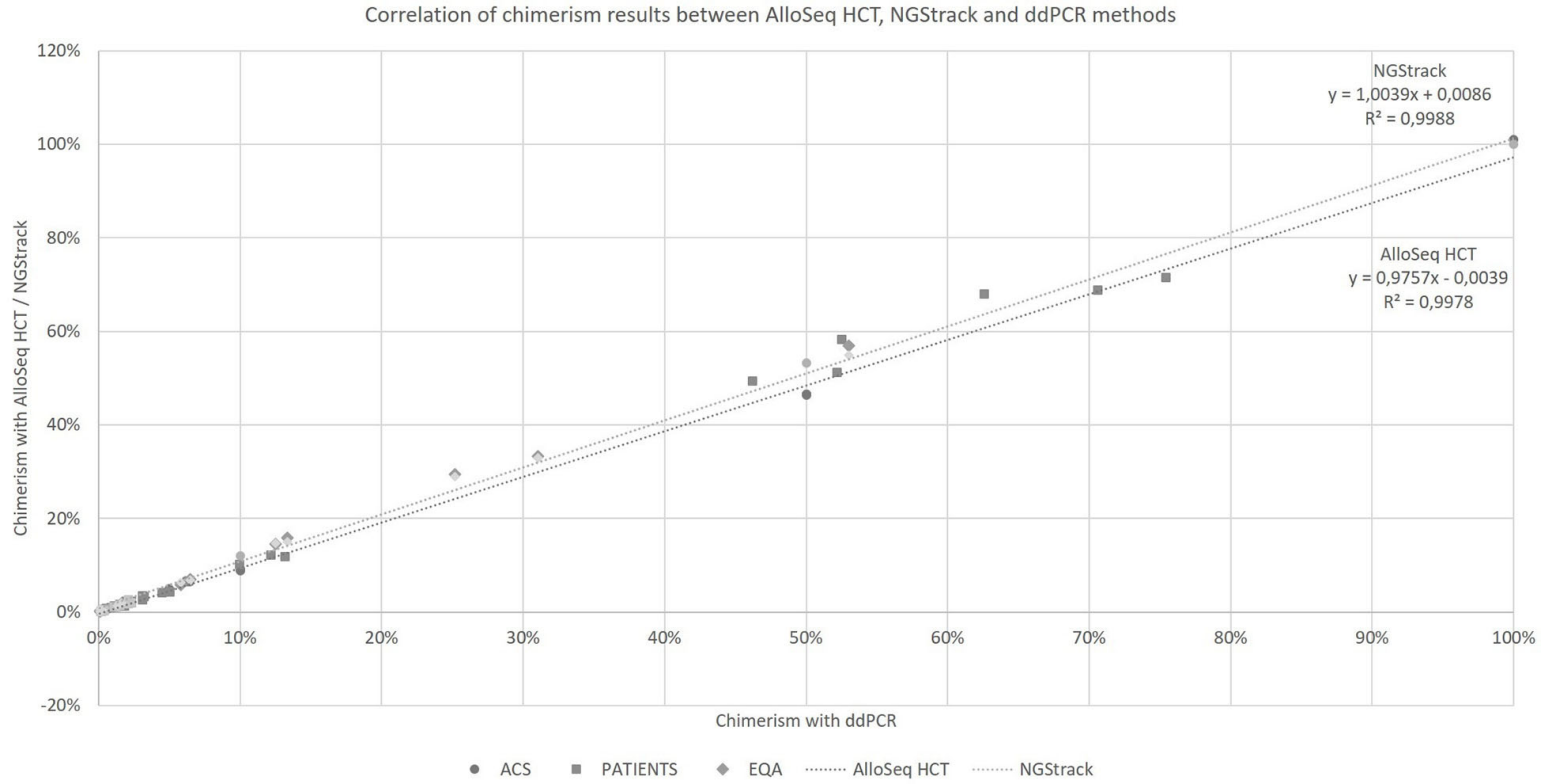
Correlation of chimerism results between AlloSeq HCT, NGStrack and ddPCR methods. ACS. Artificial Chimeric Samples; EQA. External Quality Assessment.

Taking into account all the points (details in **Supplementary Data**), regardless of the type of sample (artificial chimerism, patient or EQA) and matrix type (blood, bone marrow or sorted cells), the NGS techniques correlate with the ddPCR technique (r²=0.9978 and r²=0.9988, respectively, for AlloSeq HCT and NGStrack, **Figure 4**) over the entire range of measurement, i.e., from 0.1% to 100%.

**Figure 5:**
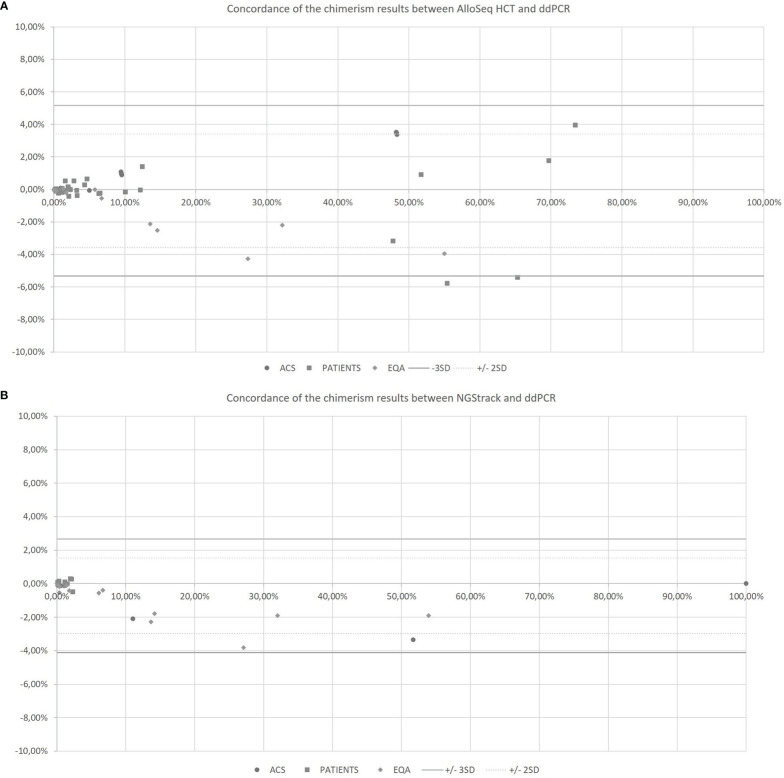
Concordance of the chimerism results between AlloSeq HCT and ddPCR **(A)** and between NGStrack and ddPCR **(B)** using the Bland-Altman plot. The Bland-Altmann plot includes the ± 2 standard deviation value (dotted lines) and ±3 standard deviation value (solid lines). ACS. Artificial Chimeric Samples; EQA. External Quality Assessment.

The AlloSeq HCT and NGStrack methods were correlated and concordant with those from the ddPCR technique using the KMRtrack kits (**Figure 5**). On a Bland−Altman plot, only one outlier (< -3 SD) was observed for the AlloSeq HCT method, but this was a point of mixed chimerism. Furthermore, all chimerism results from EQA samples (n=12) tested in both dPCR Qiacuity and dPCR Absolute Q techniques were consistent with the target results.

**Figure 6:**
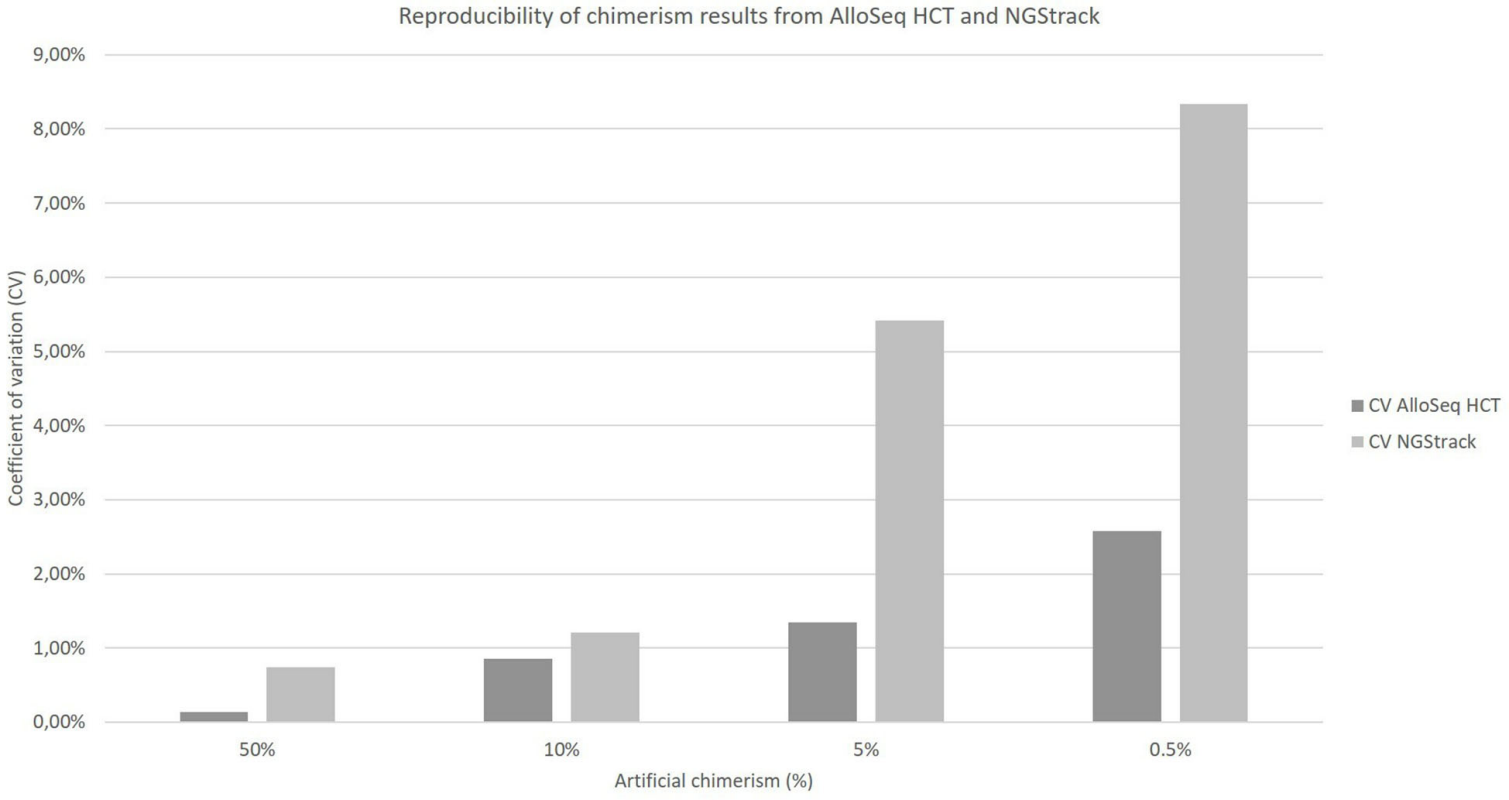
Reproducibility of chimerism results from AlloSeq HCT and NGStrack. Each chimerism percentage (50%, 10%, 5%, 0.5%) was tested on at least three different experiments to calculate the coefficient of variation of each method.

The coefficients of variation of reproducibility for both the AlloSeq HCT and NGStrack techniques are shown in **Figure 6**. The coefficients of variation for the AlloSeq HCT method were lower than those for the NGSTrack method, regardless of the chimerism percentage. These results are consistent with the limits of quantification of accuracy indicated by the suppliers, that we confirm. Indeed, the sensitivities for the AlloSeq HCT and NGStrack methods were 0.3% and 0.5%, respectively.

## Discussion

This overview of chimerism quantification techniques and methods shows that there are major innovations in the field. Although the majority of laboratories [87% (1)] still use STR, which is considered the gold standard technology, other laboratories are equipped with innovative technologies. In fact, in the current context, simple, automatable, and serial technologies are preferred. Moreover, with new applications, such as microchimerism (28), with new clinical practices, such as microtransplantation (29), and with new types of low concentration samples, such as cfDNA (30), a very good sensitivity is increasingly needed.

dPCR represents an important innovation in chimerism monitoring, as it combines the advantages of STR-PCR and qPCR (i.e., high accuracy and reproducibility and increased sensitivity) (19, 31). Some authors even have reported superior sensitivity to qPCR (20). It is an absolute quantification method, so it is not necessary to test a pretransplant sample, and the amount of DNA required is very low, thus allowing the quantification of chimerism in an aplastic patient or from cfDNA (ctDNA, cffDNA or dd-cfDNA) (30).

Both ddPCR and cdPCR techniques are perfectly transposed from the qPCR protocol and markers to dPCR, except maybe for dPCR Qiacuity for which a minor adaptation of the PCR protocol is needed. Nevertheless, the genotyping step and the selection of informative markers are always necessary in advance. The new dPCR technologies have a more important number of fluorescences (5 for dPCR Qiacuity and dCPR Absolute Q), allowing genotyping marker multiplexing amplification, as we have shown for the Crystal dPCR technique (22).

Until now, there has been no small automatic dPCR instrument. Now, the different steps of partitioning cdPCR, amplification and reading are automated in the same instrument (32), reducing run time and manual steps that are a source of error. Furthermore, they allow a homogeneous partitioning in volume that is reproducible in number (26,000 for dPCR Qiacuity and 20,480 for dPCR Absolute Q). This is a major advance for the integration of this technology in diagnostic laboratories. We have already shown that ddPCR-based chimerism works (21, 22), and in this work, we show that cdPCR systems can be used for the quantification of chimerism in a sensitive and reliable method. Thus, our study highlights that the sensitivity of cdPCR is similar to that of other dPCRs (i.e., 0.1%).

Regarding software, all systems require powerful computer equipment. Although cdCPR software is increasingly adapted to routine use in medical laboratories (especially for the dPCR Absolute Q software), it is not very adapted to the application of chimerism. Paradoxically, the Biorad software is the only one that allows the automatic calculation of the ratio illustrating chimerism. Most likely, developments are needed either by the dPCR suppliers or by the manufacturers of dPCR kits (Jeta Molecular with DigitalTrace, Biotype with DigitalQuant or Imegen with Imegen-Chimera dPCR, for example).

At the time of this publication, other innovations are in progress. First, ddPCR techniques have evolved. For example, Stilla proposed a 6-color dPCR Naica, and Biorad proposed the QXONE, a fully automated multicolor automaton. Second, the Continuum dPCR system (originally Dropworks and recently acquired by Bio-Rad Laboratories, Inc.) is a technical innovation. This digital flow PCR (dfPCR) is a continuous flow system in which samples are loaded and processed sequentially. Continuum is also an integrated instrument, which means that droplet partitioning - up to 30,000 droplets per sample - as well as thermal cycling and analysis are all performed in the same instrument. The Roche Society proposed another evolution. This is a method to perform dPCR using digital microfluidic technology in which a single consumable chip (PDMS) is fabricated to perform sample preparation, partitioning, PCR amplification, and signal detection. This technology would allow the instrument to create 20,000 events and perform amplification and melting curve generation in 7.0 min.

The emergence of next-generation sequencing has opened the possibility to develop many applications, including the quantification of chimerism by NGS. The difference with PCR techniques is to use a panel of polymorphisms (SNPs or INDELs) instead of one or two informative markers. This eliminates the need for laboratory management of all markers and allows genotyping and monitoring to be performed at the same time. Our comparison of NGS chimerism methods reveals that the three existing kits, Dvysr^®^ (Devyser Chimerism) in our previous study (22) and CareDX^®^ (AlloSeq HCT) and GendX^®^ (NGStrack) in this study are notably similar in terms of analytical performance and have a result delivery time not compatible with the emergency. However, small differences exist that are technical and linked to the software. First, the marker panels are variable from 24 to 202 markers, and it is necessary to perform genotyping for all contributors, except for AlloSeq HCT, which proposes an analysis in the absence of a contributor. On the one hand, the high number of markers avoids false-negative results caused by chromosomal deletions in the relapse of some malignancies, but on the other hand, it has been shown that too many markers are unnecessary (33). Thus, depending on the type of graft, it is necessary to test at least 20 markers if the HSCT is unrelated and at least 40 markers if it is a related HSCT. Very interestingly, NGStrack has the ability to combine chimerism analysis with other NGS analyses, such as HLA typing or HLA loss testing, and the markers used are the same as those used in KMRTrack qPCR. The Devyser chimerism NGS and Alloseq HCT kits are CEIVD marked, and both allow analysis with two donors. Finally, Alloseq is the easiest technique, with only one mix (*vs*. 7 for NGStrack) and one PCR (*vs*. 2 for Devyser chimerism NGS). We have previously shown and others have confirmed that quantification of chimerism by NGS has close agreement with the historical results of STR analysis but also with qPCR and ddPCR (22, 26). Our study confirms the detection limits communicated by the kit manufacturers, i.e., 0.3% for AlloSeq HCT and 0.5% for NGStrack. However, we show that for AlloSeq HCT, the sensitivity could be 0.1%. This sensitivity exceeds that of STR analysis (17, 22, 26, 34, 35) but is less than that of dPCR techniques. In addition to analytical performance, serial management and automation are key elements for NGS. Nevertheless, the result requires at least 48 h and is therefore not feasible in the context of a unit or an emergency.

Regarding software, all methods require powerful computer equipment that allows all three methods to give a quick result. However, there are some differences. For example, Advyser software is the only one allowing us to select or deselect markers of interest. Moreover, Advyser and AlloSeq HCT software have quality controls with color codes and allow us to comment on the results by adding patient follow-up events (treatment…). The AlloSeq HCT software will soon allow the integration of results directly into the laboratory’s computer system. However, Advyser and AlloSeq HCT software have already evolved several times. The TRKengine software, which is newest, should evolve into more software that is complete.

Finally, The two main interests of the chimerism study are the monitoring of engraftment and the prediction of disease relapse. For the first case, all the techniques can demonstrate that the hematopoiesis is of donor origin with the sensitivity near to 5% (36). In the second case, the chimerism detected at day +30 can guide appropriate therapeutic management. They require very sensitive techniques with sensitivity of at least 1% is required (37). All new methods achieve this target. If the two methods are compared in clinical practice, digital PCR is faster (result within the day *vs*. 72 hours), easier to use and easier to interpret than NGS. The sensitivity of chimerism quantification for all dPCR techniques is superior to that of all NGS methods, probably inducing earlier detection of relapses.

Another recent application that can use dPCR or qPCR, is the detection of HLA loss relapse, which occurs predominately in haploidentical transplants. Their frequency varies between 14% and 33% (38). HLA markers are used to specifically determine this type of relapse compared to classical relapse. This distinction allows appropriate therapeutic management, such as the selection of a new donor for retransplantation (39). Finally, the two new methods can be routinely used. Also, laboratories that use the NGS method on MiSeq for other hematological markers such as minimal residual disease or HLA histocompatibility applications do not need to invest in dPCR, unless a search for HLA loss is requested. Only NGStrack has the ability to determine HLA loss relapse. All NGS reagents and interpretation software are equal and the choice will depend more on the operating practices of the laboratories than on their difference in sensitivity. For laboratories with only dPCR, the instrument is compatible for all chimerism activities.

Moreover, this study proposes some elements to laboratories interested in developing the analysis of chimerism toward a new technology that also allows the development of new activities, such as the study of dd-cfDNA in organ transplantation. Indeed, in a review of the literature, Knight et al. (40) listed 47 studies (retrospective or prospective) on the analysis of dd-cfDNA as a biomarker of rejection in different organ transplants (kidney, liver, heart, lung, pancreas) from plasma or urine samples. The analyses performed are, on the one hand, the quantification of total cfDNA and the estimation of the percentage of dd-cfDNA by different techniques. These include ddPCR (41–45) and NGS (46, 47), which allow a reliable determination of the cfDNA level.

## Summary and concluding remarks

These new technologies for the quantification of chimerism allow the accurate quantification of mixed chimerism in the context of HSCT but also in organ transplantation. They can allow the combined analysis of chimerism from genomic DNA and cell-free DNA, which is the future of organ transplantation monitoring.

Currently, laboratories choose between historical and sufficient techniques for the quantification of chimerism or innovative technologies to meet new challenges. Whether using dPCR or NGS, the choice depends on the organization and habits of the laboratory, the type of patient recruitment, the cost of analysis and the involvement of teams in new clinical applications.

## Data availability statement

The original contributions presented in the study are included in the article/**Supplementary Material**. Further inquiries can be directed to the corresponding author.

## Author contributions

NC and SM performed the comparison analyses, CF, PP and CP supervised all analyses, PP, PM, MC and JC participated in the formatting of the results and the critical reading. All authors contributed to the article and approved the submitted version.
