# Supplementary material for: New methods for the quantification of mixed chimerism in transplantation
Source: Front Immunol. 2023 Jan 19;14:1023116. doi: 10.3389/fimmu.2023.1023116 (PMC9892455; doi:10.3389/fimmu.2023.1023116)
Supplement: Supplementary file 1 [file Table_1.docx]

| Sample type | Sample nature | Indication | Target | qPCR | ddPCR (Biorad) | Qiacuity | Absolute Q | AlloSeq HCT | NGStrack |
| --- | --- | --- | --- | --- | --- | --- | --- | --- | --- |
| ACS | DNA |  | 100.00% |  |  | 104.00% | 102.24% | 101.00% | 100.00% |
| ACS | DNA |  | 50.00% |  | 55.00% | 53.78% | 56.32% | 46.61% | 53.33% |
| ACS | DNA |  | 50.00% |  |  | 57.30% | 53.67% | 46.50% | 53.90% |
| ACS | DNA |  | 50.00% |  |  | 50.44% | 55.46% | 46.47% | 54.10% |
| ACS | DNA |  | 50.00% |  |  |  | 54.92% | 46.53% |  |
| ACS | DNA |  | 50.00% |  |  |  | 55.43% |  |  |
| ACS | DNA |  | 50.00% |  |  |  | 56.69% |  |  |
| ACS | DNA |  | 50.00% |  |  |  | 55.42% |  |  |
| ACS | DNA |  | 50.00% |  |  |  | 57.25% |  |  |
| ACS | DNA |  | 10.00% |  |  | 10.07% | 11.44% | 9.10% | 12.08% |
| ACS | DNA |  | 10.00% |  |  | 10.60% | 11.53% | 8.91% | 12.00% |
| ACS | DNA |  | 10.00% |  |  | 9.85% |  | 9.03% | 11.80% |
| ACS | DNA |  | 10.00% |  |  | 10.84% |  | 9.01% |  |
| ACS | DNA |  | 10.00% |  |  | 10.40% |  |  |  |
| ACS | DNA |  | 5.00% |  |  | 4.77% | 5.47% | 5.05% |  |
| ACS | DNA |  | 5.00% |  |  | 5.31% | 5.60% |  |  |
| ACS | DNA |  | 5.00% |  |  | 6.90% |  |  |  |
| ACS | DNA |  | 1.00% | 0.90% | 1.10% | 1.03% | 1.27% | 0.91% | 1.10% |
| ACS | DNA |  | 1.00% |  |  | 1.46% | 1.06% | 0.94% | 1.00% |
| ACS | DNA |  | 1.00% |  |  | 1.05% | 1.13% | 0.93% | 1.10% |
| ACS | DNA |  | 1.00% |  |  | 0.77% | 1.38% | 0.93% |  |
| ACS | DNA |  | 1.00% |  |  | 1.37% | 1.21% |  |  |
| ACS | DNA |  | 1.00% |  |  | 1.01% | 1.07% |  |  |
| ACS | DNA |  | 1.00% |  |  |  | 1.00% |  |  |
| ACS | DNA |  | 0.50% | 0.55% | 0.58% | 0.70% | 0.62% | 0.48% | 0.62% |
| ACS | DNA |  | 0.50% |  |  |  |  | 0.49% | 0.55% |
| ACS | DNA |  | 0.50% |  |  |  |  | 0.46% | 0.53% |
| ACS | DNA |  | 0.50% |  |  |  |  | 0.48% |  |
| ACS | DNA |  | 0.10% | 0.11% | 0.08% | 0.07% | 0.12% | 0.11%* | 0.20%* |
| ACS | DNA |  | 0.10% |  |  | 0.07% | 0.11% | 0.13%* |  |
| ACS | DNA |  | 0.10% |  |  | 0.15% | 0.10% | 0.09%* |  |
| ACS | DNA |  | 0.10% |  |  | 0.08% | 0.14% | 0.11%* |  |
| ACS | DNA |  | 0.10% |  |  |  | 0.07% |  |  |
| ACS | DNA |  | 0.10% |  |  |  | 0.06% |  |  |
| ACS | DNA |  | 0.10% |  |  |  | 0.03% |  |  |
| ACS | DNA |  | 0.10% |  |  |  | 0.13% |  |  |
| EQA | DNA |  | 1.30% | 1.10% | 1.34% | 1.25% | 1.02% | 1.29% | 1.44% |
| EQA | DNA |  | 0.10% | 0.06% | 0.10% | 0.09% | 0.05% | 0.13%* | 0.19%* |
| EQA | DNA |  | 0.40% | 0.35% | 0.30% | 0.38% | 0.36% | 0.39% | 0.48%* |
| EQA | DNA |  | 0.10% | 0.10% | 0.10% | 0.10% | 0.11% | 0.24%* | 0.63%* |
| EQA | DNA |  | 12.60% | 13.70% | 12.50% | 10.50% | 11.85% | 14.62% | 14.79% |
| EQA | DNA |  | 30.70% | >20% | 31.06% | 29.20% | 27.27% | 33.27% | 32.97% |
| EQA | DNA |  | 1.60% | 1.63% | 1.60% | 1.39% | 1.47% | 1.80% | 2.01% |
| EQA | DNA |  | 6.10% | 6.64% | 6.48% | 5.60% | 5.37% | 7.02% | 6.87% |
| EQA | DNA |  | 50.30% | >20% | 47.00% | 47.20% | 48.66% | 56.96% | 54.90% |
| EQA | DNA |  | 5.30% | 5.78% | 5.80% | 4.69% | 5.10% | 5.82% | 6.35% |
| EQA | DNA |  | 26.00% | >20% | 25.20% | 23.80% | 25.60% | 29.46% | 29.01% |
| EQA | DNA |  | 13.30% | 14.25% | 13.32% | 10.80% | 12.61% | 15.84% | 15.10% |
| PATIENT 1 | WB | unknown |  | 1.61% | 1.59% | 1.44% | 1.77% | 1.66% |  |
| PATIENT 1 | WB |  |  | 1.77% | 1.93% | 2.41% | 2.55% | 2.34% |  |
| PATIENT 1 | WB |  |  | 2.58% | 3.20% | 1.60% | 3.58% | 3.26% |  |
| PATIENT 1 | WB |  |  | 3.39% | 3.10% | 4.25% | 3.80% | 3.48% |  |
| PATIENT 1 | WB |  |  | 4.11% | 4.48% | 3.90% | 4.03% | 4.20% |  |
| PATIENT 1 | WB |  |  | 5.77% | 6.24% | 6.90% | 7.02% | 6.48% |  |
| PATIENT 1 | WB |  |  | 3.74% | 5.02% | 4.31% | 4.81% | 4.38% |  |
| PATIENT 1 | WB |  |  | 10.81% | 12.18% | 13.64% | 13.31% | 12.21% |  |
| PATIENT 1 | WB |  |  | 11.95% | 9.98% | 12.80% | 12.73% | 10.13% |  |
| PATIENT 1 | WB |  |  | 5.70% | 6.38% | 7.78% | 7.01% | 6.61% |  |
| PATIENT 2 | WB | ID |  | 4% | 3.12% | 3.00% | 2.96% | 2.59% |  |
| PATIENT 2 | WB |  |  | 14% | 13.20% | 12.00% | 9.58% | 11.79% |  |
| PATIENT 2 | BM |  |  | >20% | 49.00% | 50.63% | 54.60% | 49.37% |  |
| PATIENT 2 | WB |  |  | >20% | 52.20% | 52.87% | 58.79% | 51.28% |  |
| PATIENT 2 | WB |  |  | >20% | 62.60% | 65.30% | 60.54% | 68.00% |  |
| PATIENT 2 | CD15 |  |  | >20% | 52.50% | 51.24% | 60.28% | 58.28% |  |
| PATIENT 2 | WB |  |  | >20% | 70.60% | 68.61% | 74.69% | 68.83% |  |
| PATIENT 2 | WB |  |  | >20% | 75.40% | 78.29% | 79.17% | 71.45% |  |
| PATIENT 3 | WB | Th |  | 0.30% | 0.41% | 0.45% | 0.44% | 0.43% | 0.25%* |
| PATIENT 3 | WB |  |  | 0.25% | 0.21% | 0.15% | 0.30% | 0.31% | 0.27% |
| PATIENT 3 | WB |  |  | 0.16% | 0.16% | 0.18% | 0.14% | 0.18%* | 0.09%* |
| PATIENT 3 | WB |  |  | 0.90% | 1.11% | 0.95% | 0.96% | 1.28% | 1.21% |
| PATIENT 3 | WB |  |  | 1.10% | 1.21% | 1.19% | 1.51% | 1.14% | 1.10% |
| PATIENT 3 | WB |  |  | 1.50% | 1.52% | 1.58% | 1.54% | 1.58% | 1.53% |
| PATIENT 3 | WB |  |  | 2.00% | 2.31% | 2.18% | 2.29% | 2.32% | 2.03% |
| PATIENT 3 | WB |  |  | 2.00% | 2.12% | 2.09% | 2.21% | 2.07% | 1.82% |
| PATIENT 3 | CD3 |  |  | 2.00% | 2.10% | 2.55% | 1.23% | 1.94% | 2.58% |
| PATIENT 4 | WB | AML |  | 0.30% | 0.58% | 0.84% | 0.64% | 0.80% |  |
| PATIENT 4 | BM |  |  | 0.80% | 1.86% | 1.37% | 1.56% | 1.34% |  |
| PATIENT 4 | WB |  |  | 0.40% | 0.87% | 0.78% | 0.80% | 0.90% |  |
| PATIENT 4 | CD3 |  |  | 0.70% |  | 2.59% |  | 1.96% |  |
| PATIENT 4 | BM |  |  | 0.60% |  | 1.08% |  | 0.97% |  |
| PATIENT 4 | WB |  |  | 0.40% |  | 0.84% | 0.65% | 0.58% |  |
| PATIENT 5 | WB | AML |  | 0.70% |  | 0.96% | 1.26% | 1.23% |  |
| PATIENT 5 | CD33 |  |  | 1.30% |  | 1.22% |  | 1.60% |  |
| PATIENT 5 | BM |  |  | 0.20% | 0.28% | 0.22% | 0.23% | 0.24%* |  |
| PATIENT 5 | BM |  |  | 0.30% | 0.42% | 0.45% | 0.28% | 0.41% |  |
| PATIENT 5 | CD34 |  |  | 0.60% |  | 1.49% |  | 1.34% |  |

Supplementary data: total results

The reference technique is ddPCR (Biorad®)

ACS. Artificial Chimeric Samples; EQA. External Quality Assessment; WB. whole blood; BM. bone marrow; ID. Immunodeficiency; Th. Talassemia. AML. acute myelogenous leukemia; *<LOD
